# Molecular epidemiology, microbiological features and infection control strategies for carbapenem-resistant *Acinetobacter baumannii* in a German burn and plastic surgery center (2020–2022)

**DOI:** 10.1186/s13756-024-01459-5

**Published:** 2024-09-06

**Authors:** Marius Vital, Sabrina Woltemate, Dirk Schlüter, Nicco Krezdorn, Thorben Dieck, Khaled Dastagir, Franz-Christoph Bange, Ella Ebadi, Peter M. Vogt, Leonard Knegendorf, Claas Baier

**Affiliations:** 1 Institute for Medical Microbiology and Hospital Epidemiology, Hannover Medical School, Carl-Neuberg-Straße 1, 30625 Hannover, Germany; 2 Department of Plastic, Aesthetic, Hand and Reconstructive Surgery, Burn Center, Hannover Medical School, Carl-Neuberg-Straße 1, 30625 Hannover, Germany; 3 Department of Plastic and Breast Surgery, Roskilde University Hospital, 4000 Roskilde, Denmark; 4 Zealand University Hospital, Køge, Denmark

**Keywords:** *Acinetobacter baumannii*, Carbapenem, Resistance, Infection prevention and control, Epidemiology, Whole-genome sequencing, Microbiology

## Abstract

**Background:**

Carbapenem-resistant *Acinetobacter baumannii* (CRAB) frequently causes both healthcare-associated infections and nosocomial outbreaks in burn medicine/plastic surgery and beyond. Owing to the high antibiotic resistance, infections are difficult to treat, and patient outcomes are often compromised. The environmental persistence capability of CRAB favors its transmission in hospitals. A comprehensive analysis and understanding of CRAB epidemiology and microbiology are essential for guiding management.

**Methods:**

A three-year retrospective cohort study (2020–2022) was conducted in a German tertiary burn and plastic surgery center. In addition to epidemiological analyses, microbiological and molecular techniques, including whole-genome sequencing, were applied for the comprehensive examination of isolates from CRAB-positive patients.

**Results:**

During the study period, eight CRAB cases were found, corresponding to an overall incidence of 0.2 CRAB cases per 100 cases and an incidence density of 0.35 CRAB cases per 1000 patient-days. Six cases (75%) were treated in the burn intensive care unit, and four cases (50%) acquired CRAB in the hospital. Molecular analyses comprising 74 isolates supported the epidemiologic assumption that hospital acquisitions occurred within two separate clusters. In one of these clusters, environmental CRAB contamination of anesthesia equipment may have enabled transmission. Furthermore, molecular diversity of CRAB isolates within patients was observed.

**Conclusions:**

CRAB can pose a challenge in terms of infection prevention and control, especially if cases are clustered in time and space on a ward. Our study demonstrates that high-resolution phylogenetic analysis of several bacterial isolates from single patients can greatly aid in understanding transmission chains and helps to take precision control measures.

**Supplementary Information:**

The online version contains supplementary material available at 10.1186/s13756-024-01459-5.

## Background

*Acinetobacter baumannii* is a Gram-negative, nonfermenting bacterium with high environmental persistence [1, 2]. Carbapenem resistance in *Acinetobacter baumannii* is enabled mainly by carbapenemases, such as blaOXA-23 [3]. Both carbapenem-resistant *Acinetobacter baumannii* (CRAB) and susceptible isolates are well known for their ability to cause both healthcare-associated infections and nosocomial outbreaks [4–10]. Common infections caused by *Acinetobacter* species are bloodstream infections, pneumonia and soft tissue infections [11]. CRAB is listed within the priority 1 group (critical) of the WHO list of antibiotic-resistant bacteria recommended for prioritization for new antibiotics research and development [12]. In summary, CRAB represents a relevant global infection prevention and control (IPC) challenge. For these reasons, our hospital has implemented a comprehensive general IPC strategy for highly resistant Gram-negative bacteria, including CRAB [13], based on national and international recommendations. CRAB plays a particularly prominent role in burn medicine [14–16]. Patients with extensive skin and soft tissue damage are especially susceptible to infection and colonization with CRAB. In addition, burn medicine involves the frequent application of medical procedures that are associated with a high risk of environmental contamination, including extensive dressing changes and hydrotherapeutic applications.

Owing to the unfavorable resistance spectrum, the therapy of infections caused by CRAB is challenging [17]. Promising new therapeutic options, such as sulbactam/durlobactam or cefiderocol, have recently come into focus [18].

In this context, detailed epidemiological knowledge is highly useful for optimizing the IPC management of CRAB. Therefore, we conducted a comprehensive retrospective epidemiologic, microbiologic and molecular analysis of CRAB in our plastic surgery and burn medicine department (other departments were not considered) from 2020–2022.

## Methods

### Setting, study design, data acquisition and definitions

A retrospective study was conducted in the Department of Plastic, Aesthetic, Hand and Reconstructive Surgery at Hannover Medical School, a German university clinic with a burn center for adults. The study period was from January 2020 to December 2022 (i.e., 36 months). The data collection and analysis took place from January 2023 to April 2024. All procedures were performed in accordance with relevant laws and institutional guidelines. The ethics committee of Hannover Medical School approved this study (No. 10682_BO_K_2022).

The study included a burn intensive care unit (BICU) with six beds for adults in single rooms and a regular ward with 20 beds (two 4-bed rooms, two 3-bed rooms, two 2-bed rooms and two single rooms). The BICU included a room with a hydrotherapy tub and an adjacent operating theatre. Patient data, including microbiology results, were retrieved from the hospital information system, the laboratory information system and patient records. The hospital’s controlling department provided the total number of inpatient cases (inpatient stays) and patient-days. A CRAB case was defined as an inpatient stay in which CRAB was cultured from at least one microbiologic sample (screening and clinical samples). Acquisition was epidemiologically classified as nosocomial (hospital-acquired) when CRAB was found for the first time on day three or later of the hospital stay. The incidence of CRAB was calculated as CRAB cases per 100 inpatient cases, and the incidence density was calculated as CRAB cases per 1000 patient-days.

### Microbiologic diagnostic

Screening samples for carbapenem-resistant Gram-negative bacteria were cultured on CHROMagar™ mSuperCARBA™ (CHROMagar, Paris, France). Clinical samples were analyzed via standard liquid and solid culture media according to internal laboratory standards. Species identification was performed via matrix-assisted laser desorption/ionization time-of-flight mass spectrometry using a Vitek MS system (bioMérieux, Marcy-l’Étoile, France), and initial antimicrobial susceptibility testing (AST) was performed with a Vitek 2 system (bioMérieux, Marcy-l’Étoile, France). Carbapenem resistance was confirmed for at least the first isolate of every case by minimum inhibitory concentration (MIC) determination with the Merlin Micronaut system (Merlin Diagnostika, Bornheim-Hesel, Germany). European Committee on Antimicrobial Susceptibility Testing (EUCAST) standards were followed in the version valid for the respective year of the study (breakpoint tables v10.0 to v12.0). For this study, we also tested representative CRAB isolates for susceptibility to cefiderocol using the UMIC Cefiderocol (Bruker Daltonics, Bremen, Germany) and for the novel antimicrobial sulbactam/durlobactam [19] as described by Karlowsky et al*.* [20]. In brief, sulbactam (Thermo Fisher Scientific, Waltham, United States) was tested in twofold serial dilutions with a fixed durlobactam (Targetmol Chemicals, Boston, United States) concentration of 4 mg/L in cation-adjusted Mueller Hinton II Broth (BD, Heidelberg, Germany) following EUCAST recommendations for MIC determination (media preparation v7.0, reading guide v4.0).

Environmental samples were collected via RODAC plates (Tryptone Soya Agar with Disinhibitor, Oxoid, Wesel, Germany) and swabs (eSwab, Copan, Brescia, Italy).

### Routine infection prevention and control management of carbapenem-resistant *Acinetobacter baumannii*

In accordance with our hospital IPC standards, cases with CRAB were always assigned to single rooms. Staff wore gowns and gloves when entering the room of a CRAB-positive patient. In the BICU personal protective equipment (gloves and gowns) were mandatory in all patient rooms (due to immunocompromised patient status and for IPC reasons). Furthermore, the BICU staff always left the rooms of CRAB-positive patients via a separate corridor and re-entered the ward after changing their unit gowns. As our study took place during the coronavirus disease 2019 (COVID-19) pandemic, a universal masking (surgical masks) protocol was also in place.

As long as a patient with CRAB was hospitalized in a ward, all other patients in that ward were screened weekly for carbapenem-resistant bacteria, as described previously [13]. Moreover, admission screening for carbapenem-resistant Gram-negative bacteria and prophylactic isolation was recommended for patients who had previously been treated at hospitals outside of Germany. Room contact patients (if any) of CRAB-positive cases were preemptively isolated until negative swabs were obtained. Onsite medical staff were trained in dealing with patients with CRAB through targeted face-to-face and digital educational information provided by the IPC team on an event-related basis.

### Sequencing of isolates and bioinformatic analysis

CRAB isolates available in our local strain collection were recultured for molecular typing purposes. DNA was extracted (DNeasy PowerSoil Pro Kit, Qiagen, Venlo, Netherlands) for library preparation (Illumina DNA Prep, Illumina, San Diego, United States) and subsequent sequencing on an Illumina NovaSeq 6000 in paired-end mode (2 × 150 bp). Raw reads were processed as described previously [21]. In brief, the reads were quality filtered via fastp (v0.19.5) and assembled via SPAdes (v3.15.5). Subsequent gene calling was performed with Prokka (v1.14.6), and the average nucleotide identity (ANI) was calculated using fastANI (v1.33). A dendrogram was constructed in R (4.2.2) with dendextend (v1.17.1). SNP calling was performed via snippy (v4.6.0) at the contig level, with representatives of each clade of the dendrogram taken as references; a threshold of 30 SNPs was set to define a group. Carbapenemase-encoding genes were identified as described previously [22] using ariba (v2.14.4) and the CARD database (version July 2023). The Pasteur scheme from PubMLST.org [23] was used for multilocus sequence typing (MLST). In addition, we performed core genome multilocus sequence typing (cgMLST) analysis for *Acinetobacter baumannii* [24] with the SeqSphere + software suite (Ridom, Münster, Germany; client software version 10.0.4). Using the software’s default settings, de novo assembly with SKESA was performed, and a minimum spanning tree based on the cgMLST results was generated.

## Results

### Basic epidemiology and clinical characteristics

During the study period, 3999 cases were hospitalized in the two wards for a total of 22,796 patient-days. Of those, 694 (17.4%) cases and 4470 patient-days (19.6%) were in the BICU.

CRAB was detected in eight cases (six male and two female), corresponding to an overall incidence of 0.2 CRAB cases per 100 cases and an incidence density of 0.35 CRAB cases per 1000 patient-days. Six cases (75%) were treated in the BICU. Considering the BICU alone, we observed an incidence of 0.86 CRAB cases per 100 cases and an incidence density of 1.34 CRAB cases per 1000 patient-days. A summary overview of the CRAB cases, including the patient clinical characteristics, is shown in Table 1. One of the eight patients developed a bloodstream CRAB infection.

**Table 1:** Epidemiological and clinical characteristics of the eight cases with carbapenem-resistant *Acinetobacter baumannii*

Item	Number (percentage)
Total number of cases	8 (100%)
Female cases	2 (25%)
Nosocomial cases	4 (50%)
Cases with direct transfer from a foreign country hospital	4 (50%)
Median age in years [IQR]	54.5 [36.6–56.5]
Median length of stay in days [IQR]	27 [13.5–41.5]
Cases with stay in the BICU	6 (75%)
*Cases according to underlying disease*	
Explosion trauma and related burn injury	3 (37.5%)
Burn injury	4 (50%)
Fournier gangrene	1 (12.5%)
*Cases with positive sample sites*	
Rectal sample(s) positive	5 (62.5%)
Nasopharyngeal sample(s) positive	3 (37.5%)
Skin/wound sample(s) positive	8 (100%)
Urine sample(s) positive	2 (25%)
Respiratory sample(s) positive	5 (62.5%)
*Cases with cocarriage of other multidrug-resistant bacteria*	
Vancomycin-resistant enterococci	0 (0%)
Methicillin-resistant *Staphylococcus aureus*	1 (12.5%)
Carbapenem-resistant *Klebsiella pneumoniae*	2 (25%)
Carbapenem-resistant *Pseudomonas aeruginosa*	1 (12.5%)
*Cases with surgeries during hospital stay*	
One surgical intervention	7 (87.5%)
Two surgical interventions	6 (75%)
More than two surgical interventions	5 (62.5%)

IQR = Interquartile range; BICU = burn intensive care unit

Four cases (50%; case 2, case 5, case 6 and case 7) acquired CRAB within the hospital (nosocomial). The other four cases (case 1, case 3, case 4 and case 8) were found to be CRAB positive on admission (brought in). Those patients were all directly transferred from hospitals outside Germany (two patients from Poland and one patient each from Romania and Ukraine).

### Infection prevention and control measures, clusters and environmental samples

**Fig. 1:**
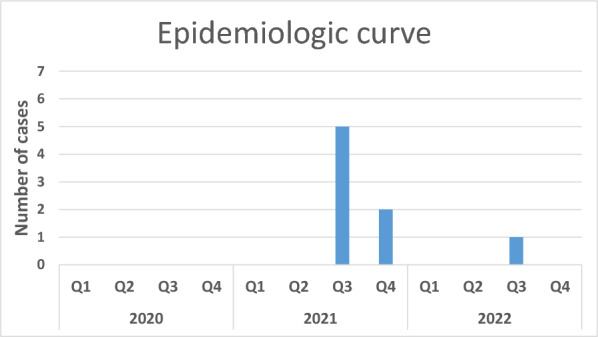
Epidemiologic curve of cases with carbapenem-resistant *Acinetobacter baumannii*

Figure 1 (epidemiologic curve) shows the distribution of the eight CRAB cases over the study period, demonstrating an increase in the number of cases in the 3rd and 4th quarters of 2021. Given the epidemiological situation at that time (e.g., joint/overlapping or subsequent stay in the BICU, care by joint staff, stay in the same functional medical units), we assumed two separate transmission events (denoted as epidemiologic clusters 1 and 2). On the one hand, we suspected transmission from Case 1 (index case) to Case 2 (cluster 1). The two impacted patients had a temporal overlap in their stay in the BICU, stayed in neighboring rooms and were cared for by the same staff. These two cases also both received hydrotherapeutic care in a tub in the BICU, but environmental examinations using swabs and contact plates revealed no evidence of CRAB in the therapy tub. The situation was resolved by strengthening the existing IPC measures and training in the ward.

**Fig. 2:**
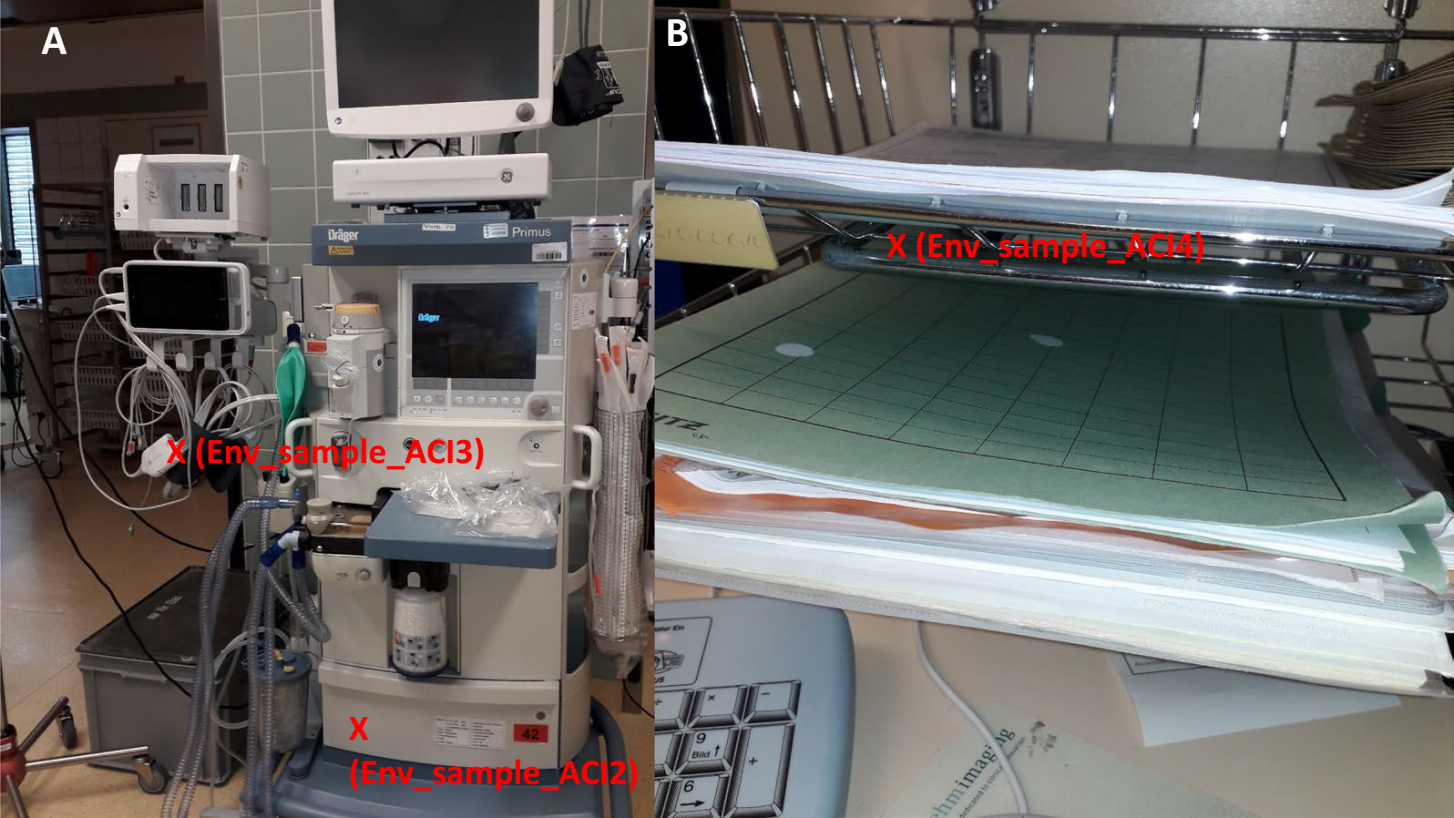
Positive environmental samples in the operating theatre. **A** Anesthesia tower in the operating theatre. **B** Documentation workplace in the operating theatre. Positive sites are marked with “X”

Second, shortly after the first transmission event, a second epidemiological cluster appeared (cluster 2) with connections to the BICU (overlapping and subsequent stays at the BICU, care provided by the same staff), consisting of cases 3, 5, 6 and 7, with case 3 most likely representing the index case. Three of these four cases were also repeatedly treated in the operating theatre next to the BICU, and CRAB was found at three environmental sample sites in the operating theatre (e.g., the anesthesia tower; see also the Phylogeny section and Fig. 2) after standard cleaning/disinfection procedures had been performed. Intensified patient screening (twice a week), intensified cleaning/disinfection in the BICU and the operating theatre, and an extensive onsite audit, training and education program for all staff (e.g., healthcare workers, cleaning staff) ended the suspected transmission chain in cluster 2. Case 4 and case 8 were not epidemiologically linked to each other or to the other cases.

### MLST and antimicrobial susceptibility

In total, 88 CRAB isolates were identified in patient samples (screening and clinical) from the department during the study period (median of seven isolates per case). Among those, 71 (80.7%) were available for sequencing. These patient isolates and the three environmental sample isolates were subjected to molecular analyses (in total, 74 isolates, see Supplementary Material 1). As a first step, we performed MLST according to the Pasteur scheme. All eight cases were colonized with ST2 isolates and case 3 was additionally colonized with ST1 isolates and ST636 isolates (see Supplementary Material 1). Table 2 shows the MIC results of selected CRAB isolates. All tested isolates were resistant to ciprofloxacin, levofloxacin, imipenem, meropenem, gentamicin and amikacin. Two isolates were susceptible or susceptible under increased exposure to trimethoprim/sulfamethoxazole; one isolate was susceptible to tobramycin, and all isolates were susceptible to colistin. All isolates but one were susceptible to cefiderocol, and all isolates but two were susceptible to sulbactam/durlobactam.

**Table 2:** Minimum inhibitory concentrations (MICs) for established antimicrobials as well as sulbactam/durlobactam for representative CRAB isolates from all cases

Strain	Case1_ACI55	Case1_ACI59	Case2_ACI68	Case3_ACI21	Case3_ACI41	Case3_ACI46	Case3_ACI33	Case3_ACI31	Case4_ACI62	Case5_ACI9	Case6_ACI73	Case7_D1	Env_ sample_ACI4	Case8_E1
Host	Case1	Case1	Case2	Case3	Case3	Case3	Case3	Case3	Case4	Case5	Case6	Case7	-	Case8
Source	Wound	Resp.	Wound	Wound	Wound	Wound	Wound	Wound	Skin (groin)	Wound	Wound	Wound	Env	Wound
MLST (Pasteur)	2	2	2	1	636	636	2	2	2	2	2	2	2	2
MIC (µg/mL)														
Ciprofloxacin	> 8 R	> 8 R	> 8 R	> 8 R	> 8 R	> 8 R	> 8 R	> 8 R	> 8 R	> 8 R	> 8 R	> 8 R	> 8 R	> 8 R
Levofloxacin	8 R	> 8 R	> 8 R	> 8 R	4 R	4 R	8 R	> 8 R	> 8 R	> 8 R	> 8 R	> 8 R	> 8 R	8 R
Imipenem	> 8 R	> 8 R	> 8 R	> 8 R	> 8 R	> 8 R	> 8 R	> 8 R	> 8 R	> 8 R	> 8 R	> 8 R	> 8 R	> 8 R
Meropenem	> 16 R	> 16 R	> 16 R	> 16 R	> 16 R	> 16 R	> 16 R	> 16 R	> 16 R	> 16 R	> 16 R	> 16 R	> 16 R	> 16 R
Trimethoprim/sulfamethoxazole	> 8 R	> 8 R	> 8 R	> 8 R	> 8 R	**4 I**	> 8 R	> 8 R	> 8 R	> 8 R	> 8 R	> 8 R	> 8 R	** ≤ 1 S**
Gentamicin^‡^	> 32 R	> 32 R	> 32 R	> 32 R	> 32 R	> 32 R	> 32 R	> 32 R	> 32 R	> 32 R	> 32 R	> 32 R	> 32 R	> 32 R
Tobramycin^‡^	> 32 R	> 32 R	> 32 R	> 32 R	> 32 R	> 32 R	> 32 R	> 32 R	> 32 R	> 32 R	> 32 R	> 32 R	> 32 R	**1 S**
Amikacin^‡^	> 32 R	> 32 R	> 32 R	> 32 R	> 32 R	> 32 R	> 32 R	> 32 R	> 32 R	> 32 R	> 32 R	> 32 R	> 32 R	> 32 R
Colistin^‡^	** ≤ 1 S**	** ≤ 1 S**	** ≤ 1 S**	** ≤ 1 S**	** ≤ 1 S**	** ≤ 1 S**	** ≤ 1 S**	** ≤ 1 S**	** ≤ 1 S**	** ≤ 1 S**	** ≤ 1 S**	** ≤ 1 S**	** ≤ 1 S**	** ≤ 1 S**
Cefiderocol^*^	**1 S**	**.25 S**	**1 S**	**.5 S**	**1 S**	**1 S**	**.25 S**	**.5 S**	**.5 S**	**.5 S**	**.5 S**	**.5 S**	**.5 S**	32 R
Sulbactam^++^	8 I	64 R	64 R	16 R	8 I	8 I	32 R	16 R	32 R	16 R	16 R	16 R	16 R	16 R
Sulbactam/durlobactam^#^	**4 S**	**4 S**	**2 S**	**1 S**	**1 S**	**2 S**	8 I	**4 S**	32 R	**4 S**	**4 S**	**4 S**	**4 S**	**4 S**

Breakpoints are according to EUCAST v13.1, unless otherwise stated. Breakpoints for antimicrobials that could be considered for treatment are printed in bold (resp. – respiratory; env. – environmental; MLST – multilocus sequence type)

^‡^If isolate is reported susceptible, use of antimicrobial can be considered in combination with another effective agent

^*^CLSI M100Ed33 Breakpoint

++ Based on CLSI M100Ed33 Breakpoint for Ampicillin/sulbactam

^#^Preliminary CLSI breakpoint: S ≤ 4/4 μg/mL; I 8/4 µg/mL; R ≥ 16/4 μg/mL. Only sulbactam concentration is shown

### Phylogenetic analysis and distribution of carbapenemase genes

**Fig. 3:**
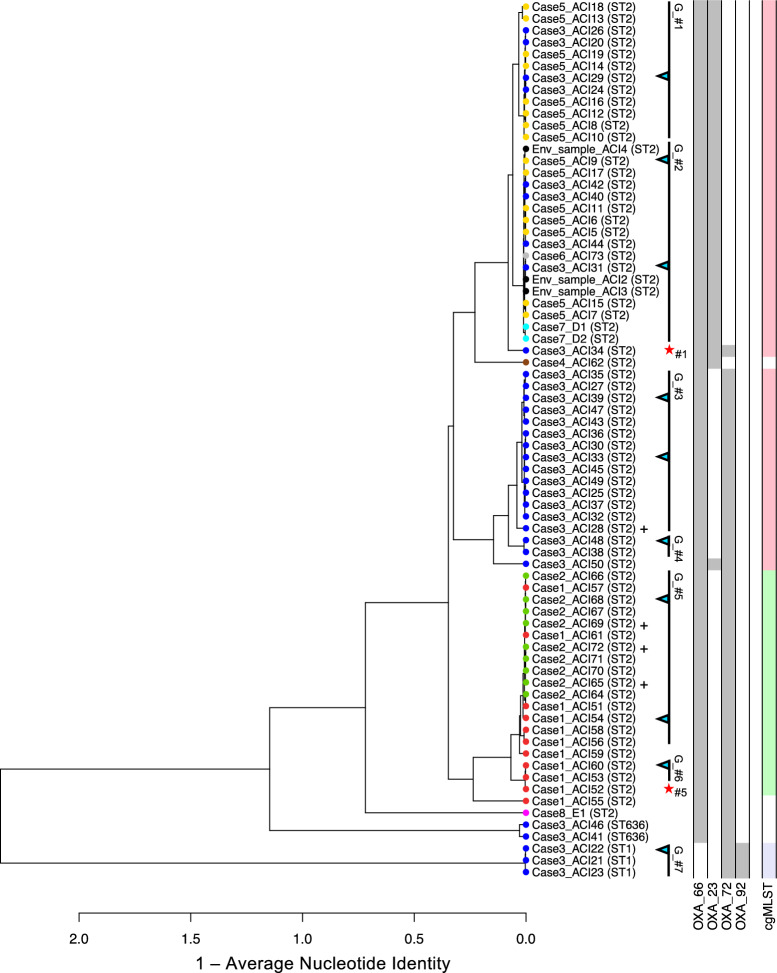
Dendrogram based on the average nucleotide identity (ANI) values of carbapenem-resistant *Acinetobacter baumannii* isolates (n = 74), along with the detected carbapenemase genes, sequence types (STs) and cgMLST results. The leaf color indicates the individual patient; environmental isolates are shown in black. Strains were grouped on the basis of their phylogenetic relatedness (G_#) via ANI and SNP analysis (30 SNPs was considered the cut-off for group designation). Cyan arrows show references for SNP calling; red stars signify deviations of SNP results (indicating the group) from ANI analysis. +: =< 35 SNPs to one reference of the group

ANI and SNP analyses revealed seven distinct groups (G_#1 to G_#7) encompassing phylogenetically closely related isolates each (Fig. 3). In accordance with the results from the MLST analyses presented above, all of the ST2 isolates clustered closely together in the dendrogram, except for E1, which displayed lower nucleotide similarity to the other strains of this group. The ST636 and ST1 isolates formed separate clades; however, the ANI values were still well within the species boundary of 95% similarity, confirming that all isolates were *Acinetobacter baumannii*. SNP analyses largely confirmed the clustering determined on the basis of ANIs, except for ACI_34, which was linked to group G_#1 by SNP analysis, but clustered separately by ANI analysis. This discrepancy is probably due to the acquisition of additional genomic material in ACI_34, which is supported by the detection of blaOXA-72 in ACI_34 but no other G_#1 member (see below). ACI_52 was assigned to G_#5 (22 and 29 SNPs to references, respectively); however, it displayed only 35 SNPs to the reference of G_#6. Group G_#5 comprised isolates from the cases 1 and 2 (cases in epidemiologic cluster 1). Group G_#2 comprised isolates from the cases in epidemiologic cluster 2 (Cases 3, 5, 6 and 7), including the three CRAB-positive environmental samples. In addition, several isolates from Cases 3 and 5 (which were part of cluster 2) clustered within another group (G_#1). Case 1, Case 3 and Case 5 carried phylogenetically distinct CRAB isolates, indicating intrahost diversity of CRAB strains.

Additionally, a cgMLST analysis was performed to add another molecular method frequently used for epidemiological analysis. The minimum spanning tree based on the cgMLST analysis was largely congruent with the ANI based results (Fig. 3); a detailed analysis (minimum spanning tree) is shown in Supplementary Material 2.

For all isolates, genes encoding carbapenemases were detected, explaining their resistance phenotypes (Fig. 3). However, the individual enzyme types differed between groups. All isolates except those linked to G_#7 (ST1) exhibited blaOXA-66. The isolates in G_#1 and G_#2, along with ACI_34, ACI_62 and ACI_50, additionally contained blaOXA-23. The strains in groups G_#3 – G_#7 possessed genes encoding blaOXA-72. Notably, all three OXA types were detected in ACI_34 and ACI_50. The strains in G_#7 (ST1) also presented genes associated with blaOXA-92.

## Discussion

In the present retrospective study of the Department of Plastic, Aesthetic, Hand and Reconstructive Surgery covering the years 2020–2022, we detected a higher incidence (0.2 CRAB cases per 100 cases in the entire department, 0.86 CRAB cases per 100 cases in the BICU) than did a previous study covering the years 2015–2019, where an overall CRAB incidence of 0.019 CRAB cases per 100 inpatient cases was observed in our entire hospital [13]. This was mainly attributable to two transmission clusters and there was no change in the overarching IPC concept compared with our prior study. An Australian study focusing on a BICU reported a higher incidence density, 3.3 per 1000 patient days from July 2019 to June 2020 [25]. In addition, studies from the U.S., South Africa, India and Singapore reported high numbers of infections caused by *Acinetobacter baumannii*, including carbapenem-resistant isolates [26–29], emphasizing the global importance of this pathogen.

The literature describes hospital clusters/outbreaks caused by CRAB in burn medicine [7, 30] and in other settings [10, 31]. High-resolution molecular analyses are helpful in providing support for epidemiologically suspected transmission clusters [4, 10]. In our case, the epidemiological assumption (two separate clusters) was confirmed by the molecular typing methods used (applying ANI and SNP analyses as well as cgMLST). We performed analyses based on ANI and SNPs to deduce the phylogenetic relationship between strains and obtain detailed insights at the whole-genome level. Furthermore, cgMLST, which represents a standardized method for investigating epidemiological events, was effective for understanding the relevant transmission patterns. Notably, within the two epidemiologically defined clusters (cluster 1 and 2), cases (patients) with genetically different CRAB isolates were found (e.g., within case 3). This emphasizes the necessity of investigating multiple isolates (for example, those collected at different time points or at different sample sites) from patients to draw appropriate conclusions and fully comprehend the overall picture of transmission.

In principle, rigorous and timely interventions to interrupt transmission chains are necessary from an IPC perspective. Some reported CRAB outbreaks required harsh measures, such as temporary closure of the affected ward, to stop the outbreak [7, 32]. The fact that environmental contamination may also have facilitated individual transmission events in the present work underlines the importance of thorough cleaning/disinfection.

Importantly, adherence to basic IPC measures (such as hand hygiene, thorough cleaning/disinfection [14] and correct handling of medical devices) is crucial for controlling CRAB in endemic and epidemic contexts, as previously reported [31]. Notably, in our case, transmission events occurred despite the use of comprehensive IPC measures and hygiene-supportive framework conditions, such as general single-room accommodations in the BICU. We have therefore strengthened adherence to known and established IPC measures through repeated training sessions and audits by the IPC team, an intervention that has been highlighted previously in a CRAB outbreak in a Swedish burn center [14].

As described by others [32], we also observed the introduction of CRAB by patients who were previously treated in hospitals outside Germany. Admission screening and prophylactic isolation for transferred patients are cornerstones to address this challenge and should be implemented.

In the event of an infection, CRAB is difficult to treat. Reserve antibiotics such as cefiderocol or colistin may be important therapeutic options in such cases [33–35]. The antimicrobial resistance tests carried out on selected isolates in this study showed broad susceptibility to these substances. Every isolate was susceptible to at least two antimicrobial substances that have been approved for use in Germany. As recent studies from India have reported resistance to colistin [36, 37] and colistin resistance has also been observed in ST2 (Pasteur) isolates in the U.S. [38], novel antibiotics such as sulbactam/durlobactam have gained importance and have been shown to be effective against CRAB infections [39]. Notably, durlobactam decreased the MIC of sulbactam by at least twofold compared with sulbactam alone, except for the isolate Case4_ACI62 in our study. One possible explanation for the phenotype of Case4_ACI62, is that the previously reported single-nucleotide polymorphism A515V in penicillin-binding protein 3 (PBP3) [40, 41] was identified in this isolate. According to a previous case report, sulbactam/durlobactam was effectively used in a critically ill burn patient [42].

In some cases, we identified isolates with differing antimicrobial susceptibility phenotype within the same patient. This may support the need for repeated MIC testing of clinically relevant CRAB isolates for a broad range of antimicrobial substances via high-quality methods.

The molecular (Pasteur sequence type) distribution of the isolates in our study is comparable to those found in Europe in the recent overview of global CRAB epidemiology by Müller et al*.* [43]. Notably, our three ST1 isolates (all from case 3) carry a blaOXA-92 (OXA-51-like) variant, which is not represented in the global collection analyzed in that study [43].

This study has several limitations. First, we collected data retrospectively and focused on a single burn and plastic surgery department, which may limit the generalizability of our findings to other healthcare settings (e.g., other medical specialties, different countries, other types of hospitals). However, investigating the epidemiology of CRAB in our entire hospital, including all departments, was beyond the scope of this study. Another limitation concerns the environmental sampling methods used in this study. Specifically, common contact plates and swabs were used and we did not use more sensitive sampling methods, such as premoistened sponge swabs (e.g., the commercially available product POLYWIPE™ Range, Medical Wire & Equipment, Corsham, England) or other comparable products. This could have led to a lower recovery rate in the environmental samples.

## Conclusions

The present study shows that in a country such as Germany, which has a comparatively low prevalence of carbapenem resistance within Gram-negative bacteria, CRAB can still occur and may be challenging in terms of IPC, particularly when clusters occur on a ward. We demonstrated that analyzing many available bacterial isolates from the same patients is an advantage for understanding transmission chains, as it allows the elucidation of potential intrahost diversity, whereas analyses of single isolates might mask transmission events. Furthermore, in our case, environmental sampling was useful for CRAB management. Finally, our results indicate that CRAB will continue to be an IPC challenge in burn medicine and beyond.

## Supplementary Information

Supplementary Material 1

Supplementary Material 2

## Data Availability

The patient data used in this study are confidential in accordance with the German Data Privacy Act, the ethics committee and the data protection commissioner of Hannover Medical School. Patient-related data such as date of ward admission, age, sex, underlying disease or length of stay are indirect identifiers that might enable patient identification. To protect patient confidentiality and participant privacy, the data used for this study can be obtained in anonymized and condensed form only, according to the Data Privacy Act. Interested researchers who meet the criteria for access to confidential data may contact the data protection commissioner of the Hannover Medical School (datenschutz@mh-hannover.de) and one of the corresponding authors (e.g., baier.claas@mh-hannover.de) to obtain access to anonymized data, as approved by the data protection commissioner and the ethics committee of the Hannover Medical School.

## Abbreviations

ANI: Average nucleotide identities
AST: Antimicrobial susceptibility testing
cgMLST: Core genome multilocus sequence typing
BICU: Burn intensive care unit
CLSI: Clinical and Laboratory Standards Institute
CRAB: Carbapenem-resistant *Acinetobacter baumannii*
IPC: Infection prevention and control
IQR: Interquartile range
MIC: Minimum inhibitory concentration
MLST: Multilocus sequence typing
ST: Sequence type

## References

[1] Wendt C, Dietze B, Dietz E, Rüden H. Survival of Acinetobacter baumannii on dry surfaces. J Clin Microbiol. 1997;35:1394–7. 10.1128/jcm.35.6.1394-1397.1997. 9163451 10.1128/jcm.35.6.1394-1397.1997 PMC229756

[2] Ng DHL, Marimuthu K, Lee JJ, Khong WX, Ng OT, Zhang W, et al. Environmental colonization and onward clonal transmission of carbapenem-resistant *Acinetobacter baumannii* (CRAB) in a medical intensive care unit: the case for environmental hygiene. Antimicrob Resist Infect Control. 2018;7:51. 10.1186/s13756-018-0343-z. 29644052 10.1186/s13756-018-0343-z PMC5891964

[3] Hamidian M, Nigro SJ. Emergence, molecular mechanisms and global spread of carbapenem-resistant *Acinetobacter baumannii*. Microb Genom. 2019;5:e000306. 10.1099/mgen.0.000306. 31599224 10.1099/mgen.0.000306 PMC6861865

[4] Makke G, Bitar I, Salloum T, Panossian B, Alousi S, Arabaghian H, et al. Whole-genome-sequence-based characterization of extensively drug-resistant *Acinetobacter baumannii* Hospital Outbreak. mSphere. 2020;5:e00934-19. 10.1128/mSphere.00934-19. 31941816 10.1128/mSphere.00934-19 PMC6968657

[5] Ben-Chetrit E, Wiener-Well Y, Lesho E, Kopuit P, Broyer C, Bier L, et al. An intervention to control an ICU outbreak of carbapenem-resistant *Acinetobacter baumannii*: long-term impact for the ICU and hospital. Crit Care. 2018;22:319. 10.1186/s13054-018-2247-y. 30463589 10.1186/s13054-018-2247-y PMC6249923

[6] Gramatniece A, Silamikelis I, Zahare I, Urtans V, Zahare I, Dimina E, et al. Control of *Acinetobacter baumannii* outbreak in the neonatal intensive care unit in Latvia: whole-genome sequencing powered investigation and closure of the ward. Antimicrob Resist Infect Control. 2019;8:84. 10.1186/s13756-019-0537-z. 31143444 10.1186/s13756-019-0537-z PMC6532256

[7] Munier A-L, Biard L, Rousseau C, Legrand M, Lafaurie M, Lomont A, et al. Incidence, risk factors, and outcome of multidrug-resistant *Acinetobacter baumannii* acquisition during an outbreak in a burns unit. J Hosp Infect. 2017;97:226–33. 10.1016/j.jhin.2017.07.020. 28751010 10.1016/j.jhin.2017.07.020

[8] Hujer AM, Higgins PG, Rudin SD, Buser GL, Marshall SH, Xanthopoulou K, et al. Nosocomial outbreak of extensively drug-resistant *Acinetobacter baumannii* Isolates containing blaOXA-237 carried on a plasmid. Antimicrob Agents Chemother. 2017;61:e00797-e817. 10.1128/AAC.00797-17. 28893775 10.1128/AAC.00797-17 PMC5655091

[9] Wybo I, Blommaert L, De Beer T, Soetens O, De Regt J, Lacor P, et al. Outbreak of multidrug-resistant *Acinetobacter baumannii* in a Belgian university hospital after transfer of patients from Greece. J Hosp Infect. 2007;67:374–80. 10.1016/j.jhin.2007.09.012. 18023922 10.1016/j.jhin.2007.09.012

[10] Thoma R, Seneghini M, Seiffert SN, Vuichard Gysin D, Scanferla G, Haller S, et al. The challenge of preventing and containing outbreaks of multidrug-resistant organisms and Candida auris during the coronavirus disease 2019 pandemic: report of a carbapenem-resistant *Acinetobacter baumannii* outbreak and a systematic review of the literature. Antimicrob Resist Infect Control. 2022;11:12. 10.1186/s13756-022-01052-8. 35063032 10.1186/s13756-022-01052-8 PMC8777447

[11] Wong D, Nielsen TB, Bonomo RA, Pantapalangkoor P, Luna B, Spellberg B. Clinical and pathophysiological overview of Acinetobacter infections: a century of challenges. Clin Microbiol Rev. 2017;30:409–47. 10.1128/CMR.00058-16. 27974412 10.1128/CMR.00058-16 PMC5217799

[12] Tacconelli E, Carrara E, Savoldi A, Harbarth S, Mendelson M, Monnet DL, et al. Discovery, research, and development of new antibiotics: the WHO priority list of antibiotic-resistant bacteria and tuberculosis. Lancet Infect Dis. 2018;18:318–27. 10.1016/S1473-3099(17)30753-3. 29276051 10.1016/S1473-3099(17)30753-3

[13] Chhatwal P, Ebadi E, Schwab F, Ziesing S, Vonberg R-P, Simon N, et al. Epidemiology and infection control of carbapenem resistant *Acinetobacter baumannii* and *Klebsiella pneumoniae* at a German university hospital: a retrospective study of 5 years (2015–2019). BMC Infect Dis. 2021;21:1196. 10.1186/s12879-021-06900-3. 34837973 10.1186/s12879-021-06900-3 PMC8627082

[14] Lindblad M, Sütterlin S, Tano E, Huss F, Lytsy B. Infection control measures to stop the spread of sequence type 15 OXA-23-producing *Acinetobacter baumannii* in a Swedish Burn Center. Burns. 2022;48:1940–9. 10.1016/j.burns.2022.01.018. 35148917 10.1016/j.burns.2022.01.018

[15] Lima WG, Silva Alves GC, Sanches C, Antunes Fernandes SO, de Paiva MC. Carbapenem-resistant *Acinetobacter baumannii* in patients with burn injury: a systematic review and meta-analysis. Burns. 2019;45:1495–508. 10.1016/j.burns.2019.07.006. 31351820 10.1016/j.burns.2019.07.006

[16] Py N, Schaal J-V, Laurent M, Renner J, Donat N. Prevention of *Acinetobacter baumannii* outbreak in a military burn center. Burns. 2022;48:1273–5. 10.1016/j.burns.2022.04.027. 35618512 10.1016/j.burns.2022.04.027

[17] Tamma PD, Aitken SL, Bonomo RA, Mathers AJ, van Duin D, Clancy CJ. Infectious diseases society of america guidance on the treatment of AmpC β-lactamase-producing enterobacterales, carbapenem-resistant *Acinetobacter baumannii*, and *Stenotrophomonas maltophilia* Infections. Clin Infect Dis. 2022;74:2089–114. 10.1093/cid/ciab1013. 34864936 10.1093/cid/ciab1013

[18] Shields RK, Paterson DL, Tamma PD. Navigating available treatment options for carbapenem-resistant *Acinetobacter baumannii*-calcoaceticus complex infections. Clin Infect Dis. 2023;76:S179–93. 10.1093/cid/ciad094. 37125467 10.1093/cid/ciad094 PMC10150276

[19] El-Ghali A, Kunz Coyne AJ, Caniff K, Bleick C, Rybak MJ. Sulbactam-durlobactam: a novel β-lactam-β-lactamase inhibitor combination targeting carbapenem-resistant *Acinetobacter baumannii* infections. Pharmacotherapy. 2023;43:502–13. 10.1002/phar.2802. 37052117 10.1002/phar.2802

[20] Karlowsky JA, Hackel MA, McLeod SM, Miller AA. In Vitro Activity of sulbactam-durlobactam against global isolates of *Acinetobacter baumannii*-calcoaceticus complex collected from 2016 to 2021. Antimicrob Agents Chemother. 2022;66:e0078122. 10.1128/aac.00781-22. 36005804 10.1128/aac.00781-22 PMC9487466

[21] Chhatwal P, Woltemate S, Ziesing S, Welte T, Schlüter D, Vital M. Molecular characterization and improved diagnostics of nocardia strains isolated over the last two decades at a German tertiary care center. EXCLI J. 2021;20:851–62. 10.17179/excli2021-3787. 34177407 10.17179/excli2021-3787 PMC8222631

[22] Schäfer F, Görner P, Woltemate S, Brandenberger C, Geffers R, Ziesing S, et al. The resistance mechanism governs physiological Adaptation of *Escherichia coli* to growth with sublethal concentrations of carbapenem. Front Microbiol. 2021;12:812544. 10.3389/fmicb.2021.812544. 35173695 10.3389/fmicb.2021.812544 PMC8841762

[23] Jolley KA, Bray JE, Maiden MCJ. Open-access bacterial population genomics: BIGSdb software, the PubMLSTorg website and their applications. Wellcome Open Res. 2018;3:124. 10.12688/wellcomeopenres.14826.1. 30345391 10.12688/wellcomeopenres.14826.1 PMC6192448

[24] Higgins PG, Prior K, Harmsen D, Seifert H. Development and evaluation of a core genome multilocus typing scheme for whole-genome sequence-based typing of Acinetobacter baumannii. PLoS ONE. 2017;12:e0179228. 10.1371/journal.pone.0179228. 28594944 10.1371/journal.pone.0179228 PMC5464626

[25] Cleland H, Tracy LM, Padiglione A, Stewardson AJ. Patterns of multidrug resistant organism acquisition in an adult specialist burns service: a retrospective review. Antimicrob Resist Infect Control. 2022;11:82. 10.1186/s13756-022-01123-w. 35698209 10.1186/s13756-022-01123-w PMC9195457

[26] Keen EF, Robinson BJ, Hospenthal DR, Aldous WK, Wolf SE, Chung KK, et al. Prevalence of multidrug-resistant organisms recovered at a military burn center. Burns. 2010;36:819–25. 10.1016/j.burns.2009.10.013. 20080354 10.1016/j.burns.2009.10.013

[27] Bahemia IA, Muganza A, Moore R, Sahid F, Menezes CN. Microbiology and antibiotic resistance in severe burns patients: a 5 year review in an adult burns unit. Burns. 2015;41:1536–42. 10.1016/j.burns.2015.05.007. 26051799 10.1016/j.burns.2015.05.007

[28] Singh NP, Rani M, Gupta K, Sagar T, Kaur IR. Changing trends in antimicrobial susceptibility pattern of bacterial isolates in a burn unit. Burns. 2017;43:1083–7. 10.1016/j.burns.2017.01.016. 28153582 10.1016/j.burns.2017.01.016

[29] Chim H, Tan BH, Song C. Five-year review of infections in a burn intensive care unit: high incidence of *Acinetobacter baumannii* in a tropical climate. Burns. 2007;33:1008–14. 10.1016/j.burns.2007.03.003. 17996555 10.1016/j.burns.2007.03.003

[30] Shenoy ES, Pierce VM, Sater MRA, Pangestu FK, Herriott IC, Anahtar MN, et al. Community-acquired in name only: a cluster of carbapenem-resistant *Acinetobacter baumannii* in a burn intensive care unit and beyond. Infect Control Hosp Epidemiol. 2020;41:531–8. 10.1017/ice.2020.15. 32106898 10.1017/ice.2020.15

[31] Schlosser B, Weikert B, Fucini G-B, Kohlmorgen B, Kola A, Weber A, et al. Risk factors for transmission of carbapenem-resistant *Acinetobacter baumannii* in outbreak situations: results of a case-control study. BMC Infect Dis. 2024;24:120. 10.1186/s12879-024-09015-7. 38263063 10.1186/s12879-024-09015-7 PMC10807151

[32] Landelle C, Legrand P, Lesprit P, Cizeau F, Ducellier D, Gouot C, et al. Protracted outbreak of multidrug-resistant *Acinetobacter baumannii* after intercontinental transfer of colonized patients. Infect Control Hosp Epidemiol. 2013;34:119–24. 10.1086/669093. 23295556 10.1086/669093

[33] Onorato L, de Luca I, Monari C, Coppola N. Cefiderocol either in monotherapy or combination versus best available therapy in the treatment of carbapenem-resistant *Acinetobacter baumannii* infections: A systematic review and meta-analysis. J Infect. 2024;88:106113. 10.1016/j.jinf.2024.01.012. 38331328 10.1016/j.jinf.2024.01.012

[34] Giannella M, Verardi S, Karas A, Abdel Hadi H, Dupont H, Soriano A, et al. Carbapenem-resistant Acinetobacter spp. infection in critically ill patients with limited treatment options: a descriptive study of cefiderocol therapy during the COVID-19 pandemic. Open Forum Infect Dis. 2023;10:ofad329. 10.1093/ofid/ofad329. 37496600 10.1093/ofid/ofad329 PMC10368198

[35] Paul M, Carrara E, Retamar P, Tängdén T, Bitterman R, Bonomo RA, et al. European Society of Clinical Microbiology and Infectious Diseases (ESCMID) guidelines for the treatment of infections caused by multidrug-resistant Gram-negative bacilli (endorsed by European society of intensive care medicine). Clin Microbiol Infect. 2022;28:521–47. 10.1016/j.cmi.2021.11.025. 34923128 10.1016/j.cmi.2021.11.025

[36] Sharma S, Banerjee T, Yadav G, Kumar A. Susceptibility profile of bla OXA-23 and metallo-β-lactamases co-harbouring isolates of carbapenem resistant *Acinetobacter baumannii* (CRAB) against standard drugs and combinations. Front Cell Infect Microbiol. 2022;12:1068840. 10.3389/fcimb.2022.1068840. 36683677 10.3389/fcimb.2022.1068840 PMC9853021

[37] Rout BP, Dash SK, Otta S, Behera B, Praharaj I, Sahu KK. Colistin resistance in carbapenem non-susceptible *Acinetobacter baumanii* in a tertiary care hospital in India: clinical characteristics, antibiotic susceptibility and molecular characterization. Mol Biol Rep. 2024;51:357. 10.1007/s11033-023-08982-5. 38400950 10.1007/s11033-023-08982-5

[38] Iovleva A, Mustapha MM, Griffith MP, Komarow L, Luterbach C, Evans DR, et al. Carbapenem-resistant *Acinetobacter baumannii* in US hospitals: diversification of circulating lineages and antimicrobial resistance. mBio. 2022;13:e0275921. 10.1128/mbio.02759-21. 35311529 10.1128/mbio.02759-21 PMC9040734

[39] Kaye KS, Shorr AF, Wunderink RG, Du B, Poirier GE, Rana K, et al. Efficacy and safety of sulbactam-durlobactam versus colistin for the treatment of patients with serious infections caused by *Acinetobacter baumannii*-calcoaceticus complex: a multicentre, randomised, active-controlled, phase 3, non-inferiority clinical trial (ATTACK). Lancet Infect Dis. 2023;23:1072–84. 10.1016/S1473-3099(23)00184-6. 37182534 10.1016/S1473-3099(23)00184-6

[40] Findlay J, Poirel L, Bouvier M, Nordmann P. In vitro activity of sulbactam-durlobactam against carbapenem-resistant *Acinetobacter baumannii* and mechanisms of resistance. J Glob Antimicrob Resist. 2022;30:445–50. 10.1016/j.jgar.2022.05.011. 35618210 10.1016/j.jgar.2022.05.011

[41] McLeod SM, Moussa SH, Hackel MA, Miller AA. In vitro activity of sulbactam-durlobactam against *Acinetobacter baumannii*-calcoaceticus complex isolates collected globally in 2016 and 2017. Antimicrob Agents Chemother. 2020;64:e02534-e2619. 10.1128/AAC.02534-19. 31988095 10.1128/AAC.02534-19 PMC7179289

[42] Tiseo G, Giordano C, Leonildi A, Riccardi N, Galfo V, Limongi F, et al. Salvage therapy with sulbactam/durlobactam against cefiderocol-resistant *Acinetobacter baumannii* in a critically ill burn patient: clinical challenges and molecular characterization. JAC Antimicrob Resist. 2023;5:dlad078. 10.1093/jacamr/dlad078. 37325251 10.1093/jacamr/dlad078 PMC10265591

[43] Müller C, Reuter S, Wille J, Xanthopoulou K, Stefanik D, Grundmann H, et al. A global view on carbapenem-resistant *Acinetobacter baumannii*. mBio. 2023;14:e0226023. 10.1128/mbio.02260-23. 37882512 10.1128/mbio.02260-23 PMC10746149

