# Supplementary material for: Molecular epidemiology, microbiological features and infection control strategies for carbapenem-resistant Acinetobacter baumannii in a German burn and plastic surgery center (2020–2022)
Source: Antimicrob Resist Infect Control. 2024 Sep 6;13:99. doi: 10.1186/s13756-024-01459-5 (PMC11378564; doi:10.1186/s13756-024-01459-5)
Supplement: Supplementary file 1 — Supplementary Material 1 [file 13756_2024_1459_MOESM1_ESM.docx]

**Supplementary Material 1.** Overview of the carbapenem-resistant *Acinetobacter baumannii* isolates analyzed in the study.

| **Case** (acquisition category*) | **Isolate label** (strain) | **Sampling date** (yyyy-mm-dd) | **MLST result** (Pasteur) |
| --- | --- | --- | --- |
| 1  (brought in) | Case1_ACI51 | 2021-07-30 | 2 |
|  | Case1_ACI52 | 2021-07-30 | 2 |
|  | Case1_ACI53 | 2021-07-30 | 2 |
|  | Case1_ACI54 | 2021-07-30 | 2 |
|  | Case1_ACI55 | 2021-07-30 | 2 |
|  | Case1_ACI56 | 2021-07-30 | 2 |
|  | Case1_ACI57 | 2021-08-04 | 2 |
|  | Case1_ACI58 | 2021-08-06 | 2 |
|  | Case1_ACI59 | 2021-08-10 | 2 |
|  | Case1_ACI60 | 2021-08-09 | 2 |
|  | Case1_ACI61 | 2021-08-09 | 2 |
| 2  (nosocomial) | Case2_ACI64 | 2021-08-09 | 2 |
|  | Case2_ACI65 | 2021-08-11 | 2 |
|  | Case2_ACI66 | 2021-08-09 | 2 |
|  | Case2_ACI67 | 2021-08-10 | 2 |
|  | Case2_ACI68 | 2021-08-09 | 2 |
|  | Case2_ACI69 | 2021-08-09 | 2 |
|  | Case2_ACI70 | 2021-08-12 | 2 |
|  | Case2_ACI71 | 2021-08-14 | 2 |
|  | Case2_ACI72 | 2021-08-14 | 2 |
| 3  (brought in) | Case3_ACI20 | 2021-08-21 | 2 |
|  | Case3_ACI21 | 2021-08-20 | 1 |
|  | Case3_ACI22 | 2021-08-20 | 1 |
|  | Case3_ACI23 | 2021-08-20 | 1 |
|  | Case3_ACI24 | 2021-08-26 | 2 |
|  | Case3_ACI25 | 2021-08-25 | 2 |
|  | Case3_ACI26 | 2021-08-30 | 2 |
|  | Case3_ACI27 | 2021-08-30 | 2 |
|  | Case3_ACI28 | 2021-08-30 | 2 |
|  | Case3_ACI29 | 2021-09-01 | 2 |
|  | Case3_ACI30 | 2021-08-30 | 2 |
|  | Case3_ACI31 | 2021-09-06 | 2 |
|  | Case3_ACI32 | 2021-09-06 | 2 |
|  | Case3_ACI33 | 2021-09-08 | 2 |
|  | Case3_ACI34 | 2021-09-13 | 2 |
|  | Case3_ACI35 | 2021-09-13 | 2 |
|  | Case3_ACI36 | 2021-09-13 | 2 |
|  | Case3_ACI37 | 2021-09-13 | 2 |
|  | Case3_ACI38 | 2021-09-19 | 2 |
|  | Case3_ACI39 | 2021-09-20 | 2 |
|  | Case3_ACI40 | 2021-09-20 | 2 |
|  | Case3_ACI41 | 2021-09-20 | 636 |
|  | Case3_ACI42 | 2021-09-20 | 2 |
|  | Case3_ACI43 | 2021-09-27 | 2 |
|  | Case3_ACI44 | 2021-09-27 | 2 |
|  | Case3_ACI45 | 2021-09-27 | 2 |
|  | Case3_ACI46 | 2021-09-27 | 636 |
|  | Case3_ACI47 | 2021-10-04 | 2 |
|  | Case3_ACI48 | 2021-10-11 | 2 |
|  | Case3_ACI49 | 2021-10-11 | 2 |
|  | Case3_ACI50 | 2021-10-11 | 2 |
| 4  (brought in) | Case4_ACI62 | 2021-08-20 | 2 |
| 5  (nosocomial) | Case5_ACI10 | 2021-10-14 | 2 |
|  | Case5_ACI11 | 2021-10-16 | 2 |
|  | Case5_ACI12 | 2021-10-18 | 2 |
|  | Case5_ACI13 | 2021-10-18 | 2 |
|  | Case5_ACI14 | 2021-11-01 | 2 |
|  | Case5_ACI15 | 2021-11-01 | 2 |
|  | Case5_ACI16 | 2021-11-01 | 2 |
|  | Case5_ACI17 | 2021-11-01 | 2 |
|  | Case5_ACI18 | 2021-11-08 | 2 |
|  | Case5_ACI19 | 2021-11-08 | 2 |
|  | Case5_ACI5 | 2021-09-28 | 2 |
|  | Case5_ACI6 | 2021-10-01 | 2 |
|  | Case5_ACI7 | 2021-09-30 | 2 |
|  | Case5_ACI8 | 2021-10-04 | 2 |
|  | Case5_ACI9 | 2021-09-29 | 2 |
| 6  (nosocomial) | Case6_ACI73 | 2021-10-22 | 2 |
| 7  (nosocomial) | Case7_D1 | 2021-12-04 | 2 |
|  | Case7_D2 | 2021-12-04 | 2 |
| 8  (brought in) | Case8_E1 | 2022-07-27 | 2 |
| Environmental Samples | Env_sample_ACI2 | 2021-10-11 | 2 |
|  | Env_sample_ACI3 | 2021-10-11 | 2 |
|  | Env_sample_ACI4 | 2021-10-11 | 2 |

*The acquisition category (nosocomial/brought in) refers to the epidemiological classification of the respective case (see also main text).
