# Supplementary material for: Molecular epidemiology, microbiological features and infection control strategies for carbapenem-resistant Acinetobacter baumannii in a German burn and plastic surgery center (2020–2022)
Source: Antimicrob Resist Infect Control. 2024 Sep 6;13:99. doi: 10.1186/s13756-024-01459-5 (PMC11378564; doi:10.1186/s13756-024-01459-5)
Supplement: Supplementary file 2 — Supplementary Material 2 [file 13756_2024_1459_MOESM2_ESM.pdf]

Supplementary Material 2. cgMLST analysis; Minimum spanning tree (created within Ridom Seqsphere+; setting: pairwise ignore missing values) of carbapenem-resistant *Acinetobacter baumannii* isolates (n=74). Clustering distance: 9. Isolates that are assigned to the same circle have no allelic differences. cgMLST clusters are highlighted in color. The numbers next to the lines indicate the number of allelic differences (the lengths of the lines are not proportional to the allelic differences for reasons of space).

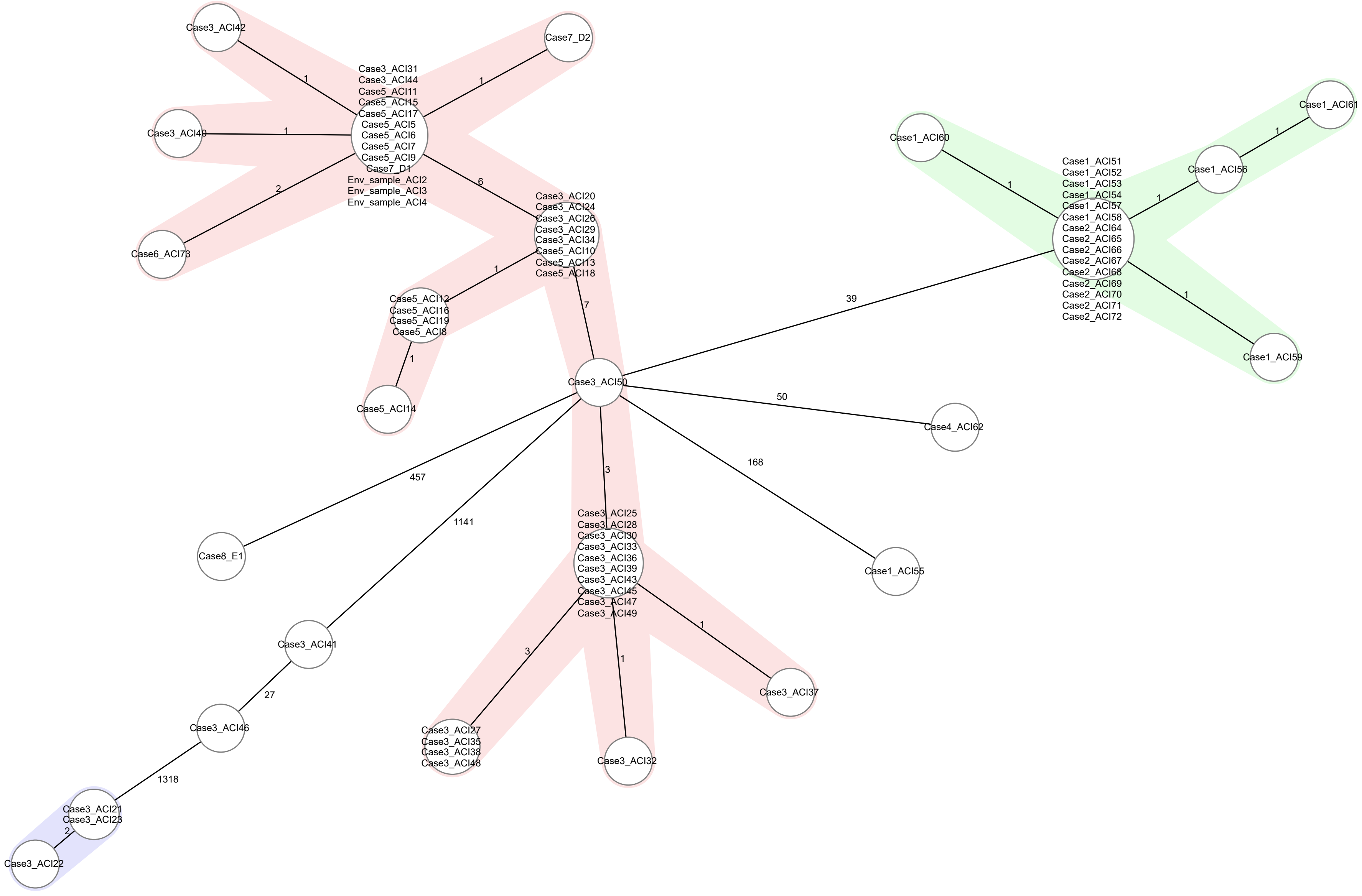

Case3_ACI42
Case7_D2
Case3_ACI31
Case3_ACI44
Case5_ACI11
Case5_ACI15
Case5_ACI17
Case5_ACI5
Case5_ACI6
Case5_ACI7
Case5_ACI9
Case7_D1
Env_sample_ACI2
Env_sample_ACI3
Env_sample_ACI4
Case3_ACI40
Case6_ACI73
Case3_ACI20
Case3_ACI24
Case3_ACI26
Case3_ACI29
Case3_ACI34
Case5_ACI10
Case5_ACI13
Case5_ACI18
Case5_ACI12
Case5_ACI16
Case5_ACI19
Case5_ACI8
Case5_ACI14
Case3_ACI50
Case1_ACI60
Case1_ACI61
Case1_ACI56
Case1_ACI51
Case1_ACI52
Case1_ACI53
Case1_ACI54
Case1_ACI57
Case1_ACI58
Case2_ACI64
Case2_ACI65
Case2_ACI66
Case2_ACI67
Case2_ACI68
Case2_ACI69
Case2_ACI70
Case2_ACI71
Case2_ACI72
Case1_ACI59
Case4_ACI62
Case8_E1
Case1_ACI55
Case3_ACI25
Case3_ACI28
Case3_ACI30
Case3_ACI33
Case3_ACI36
Case3_ACI39
Case3_ACI43
Case3_ACI45
Case3_ACI47
Case3_ACI49
Case3_ACI41
Case3_ACI46
Case3_ACI37
Case3_ACI27
Case3_ACI35
Case3_ACI38
Case3_ACI48
Case3_ACI32
Case3_ACI21
Case3_ACI23
Case3_ACI22
1
1
1
2
6
1
7
1
39
1
1
1
1
50
168
457
1141
3
3
1
1
27
1318
2
